# Chromatographic purification of small extracellular vesicles using an affinity column for phospholipid membranes

**DOI:** 10.1007/s10529-023-03430-7

**Published:** 2023-10-03

**Authors:** Kanako Masaki, Abo Bakr F. Ahmed, Takenori Ishida, Yuuki Mikami, Hisakage Funabashi, Ryuichi Hirota, Takeshi Ikeda, Akio Kuroda

**Affiliations:** 1 Unit of Biotechnology, Graduate School of Integrated Sciences for Life, Hiroshima University, 1-3-1 Kagamiyama, Higashi-Hiroshima Hiroshima, 739-8530 Japan; 2 Department of Microbiology and Immunology, Faculty of Pharmacy, Minia University, Minia, 61519 Egypt

**Keywords:** Affinity peptide, Column chromatography, Exosome, Extracellular vesicles, Purification

## Abstract

**Objectives:**

This study aimed to investigate whether chromatography using an ExoPUA column, an affinity column for phospholipid membranes, could potentially serve as an efficient, rapid, scalable, and reproducible method for purifying small extracellular vesicles (sEVs).

**Results:**

We used the ExoPUA column connected to a fast-performance liquid chromatography system. One-step chromatographic purification of sEVs from culture supernatant using the ExoPUA protocol resulted in an 82 ± 16-fold increase in purity with a yield of 38 ± 5% of sEVs. The purified sEVs contained CD9, CD63, TSG101, and miRNA (miR-21), but not the endoplasmic reticulum protein Calnexin. Transmission electron microscopy indicated that the purified sEVs were intact. The purification performance of the ExoPUA protocol showed superior results in terms of yield compared to that of the differential ultracentrifugation method, the most commonly used method for purifying sEVs in laboratories, and purity compared to that of the DEAE chromatography protocol.

**Conclusion:**

The sEVs were effectively purified in the bind-elute mode and the ExoPUA column can be refreshed and sterilized with sodium hydroxide (NaOH), having high potential for multiple sEV purification in a scalable and industrial manner.

**Supplementary Information:**

The online version contains supplementary material available at 10.1007/s10529-023-03430-7.

## Introduction

Extracellular vesicles (EVs) are lipid-bilayer vesicles that are released from virtually every cell into the extracellular space (EL Andaloussi et al. 2013; Verweij et al. 2021). Among them, small EVs (sEVs), such as exosomes, have garnered increasing interest owing to not only their diagnostic applications but also their therapeutic potential (Bruno et al. 2009; Doeppner et al. 2015; Kadota et al. 2021; Kakizaki et al. 2021; Lai et al. 2010; Takeuchi et al. 2021; Zhang et al. 2020). For example, mesenchymal stem cell (MSC)-derived sEVs recapitulate the therapeutic and regenerative effects of MSCs on damaged tissues/organs in models of myocardial ischemia (Lai et al. 2010), acute tubular injury (Bruno et al. 2009), stroke (Doeppner et al. 2015), liver fibrosis (Takeuchi et al. 2021), and acute liver failure (Zhang et al. 2020). In addition to MSC-derived sEVs, human bronchial epithelial cell-derived sEVs have been suggested to attenuate pulmonary fibrosis with more pronounced effects than those observed with MSC-derived sEVs (Kadota et al. 2021). In addition, human hepatocyte-derived sEVs have the potential to attenuate acute liver injury (Kakizaki et al. 2021).

To date, several methods for sEV purification have been developed, including those based on differential ultracentrifugation (UC), size, immunoaffinity capture, and microfluids, as well as sEV precipitation methods (Li et al. 2017; Sharma et al. 2016; Wang et al. 2021). Among these, the UC method is considered the gold standard and most commonly used method for sEV purification in laboratories. However, following differential centrifugation, sample volumes are limited, and this method cannot be readily scaled up. In addition, although the purification of structurally and biologically intact sEVs is required for their therapeutic applications, collapse and damage of EV membranes, or EV aggregation due to mechanical stress during UC, have been reported (Lee et al. 2016). Size-exclusion chromatography can potentially yield intact sEVs because using gravity flow allows for the retention of vesicle structure and integrity, preserving the biological activity of sEVs (Lozano-Ramos et al. 2015). However, size exclusion chromatography is a technique that may require concentration before and after purification. Thus, a key challenge in the therapeutic use of sEVs is developing a method to purify sEVs in a timely and cost-effective manner at the required purity and appropriate scale.

Previously, we found that peptides containing 8 or 16 lysine residues have an affinity for phospholipid membranes, with dissociation constants of 102 nM and 11.6 nM for phosphatidylserine, respectively (Ishida et al. 2020). Magnetic beads immobilized with the affinity peptides captured sEVs from a culture supernatant of MCF7 human breast cancer cells. Importantly, the bound sEVs could be dissociated from the beads under mild conditions using 0.5 M NaCl, resulting in isolated sEVs retaining their spherical shape (Ishida et al. 2020). Using these affinity beads, other groups have demonstrated that sEVs can be isolated from the cerebrospinal fluid (Sjoqvist et al. 2020). In our previous study, we adapted a pre-existing bench-top instrument for magnetic separation to perform automated sEV purification with higher purity and yield than previously obtained by using standard UC methods (Ishida et al. 2020). This automated system can process a maximum of six samples in parallel; however, sample volumes are limited to 500 µL. Therefore, other purification systems, such as column chromatography immobilized with affinity peptides, may offer alternative solutions for scalable sEV purification. One advantage of bind-elute chromatography is scalability, such as manufacturing scale columns of several hundred liters (Aldington and Bonnerjea 2007), which have the potential to meet the demands, particularly in the therapeutic use of sEVs through an efficient, rapid, scalable, and reproducible purification method (Colao et al. 2018; Xu et al. 2016). Therefore, this study aimed to investigate whether chromatography using an ExoPUA column, a cellulose-based resin column immobilized with peptides consisting of 25–35 lysine residues, could meet such demands. Specifically, we tested whether the ExoPUA column could be used for sEV purification from a culture supernatant in the bind-elute mode with appropriate purity and yield.

## Materials and methods

### Cell culture

The human breast cancer cell line MCF7 was obtained from the Japanese Collection of Research Bioresources Cell Bank (Osaka, Japan). Cells were cultured in Dulbecco’s Modified Eagle Medium (DMEM) (Thermo Fisher Scientific, Waltham, MA, USA), supplemented with 10% heat-inactivated fetal bovine serum (HyClone FBS; GE Healthcare Life Sciences, Logan, UT, USA), 100 units/mL of penicillin, and 0.1 mg/mL of streptomycin in a humidified incubator with 95% air and 5% CO_2_ at 37 °C. After 48 h (cells reached a confluency of ~ 80%), the medium was changed to a serum-free synthetic medium (advanced DMEM, Thermo Fisher Scientific) followed by a 48-h incubation. The culture medium was collected and centrifuged at 2000 × *g* for 10 min, followed by centrifugation at 10,000 × *g* for 30 min. The resulting supernatant was passed through a 0.2-μm membrane filter and stored at − 80 °C as a culture supernatant of MCF7 cells.

### Isolation of sEVs using an ExoPUA column

A 1-mL ExoPUA column (Siliconbio Inc., Hiroshima, Japan) was connected to an ÄKTA Purifier 10 system (Cytiva, Marlborough, MA, USA) and pre-equilibrated with A buffer (10 mM sodium phosphate buffer at pH 8.0 containing 100 mM NaCl and 0.005% Tween 20). All experiments in this study were performed using the 1-mL ExoPUA column. The MCF7 culture supernatant and one-tenth volume of 550 mM MES buffer (pH 5.5) containing 0.055% Tween 20 were mixed and applied to the ExoPUA column at a flow rate of 1 mL/min. As the inner diameter of the column is 6.7 mm, the linear flow rate is calculated as 170 cm/h. After sample injection, the column was washed with A buffer until A_280_ reached baseline. One-milliliter fractions containing sEVs were obtained by a linear gradient of 6 mL of B buffer (10 mM sodium phosphate buffer (pH 8.0) containing 1 M NaCl and 0.005% Tween 20). To refresh the column, 2 mL of 0.2 M NaOH was injected into the column and allowed to stand overnight at around 18 °C. The column was washed with 10 mL of 20% ethanol, stored at 4 °C, and used repeatedly. For the experiment using phospholipid membranes, liposomes composed of dipalmitoylphosphatidylcholine (DPPC), dipalmitoylphosphatidylserine (DPPS), and cholesterol (5:1:4, molar ratio) (Katayama Chemical, Japan) were used instead of the MCF7 culture supernatant.

### UC method

The sEVs were obtained by sedimentation using UC (Livshits et al. 2015; Théry et al. 2006). Four milliliters of the MCF7 culture supernatant were centrifuged at 100,000 × *g* for 70 min at 4 °C using an Optima TLX with a TLA100.3 fixed-angle rotor (Beckman Coulter, Brea, CA, USA). The supernatant was discarded, and the pellet was resuspended in PBS (Nacalai Tesque, Kyoto, Japan). The mixture was re-centrifuged at 100,000 × *g* for 70 min at 4 °C, and the pellet was resuspended in 1 mL of PBS.

### Diethylaminoethyl (DEAE) method

A 1-mL DEAE-Sepharose FF anion exchange column (Cytiva) was connected to an ÄKTA Purifier 10 system. The diameters of the DEAE-Sepharose are 45–165 µm, a range similar to that of ExoPUA resin (40–130 µm). The column was pre-equilibrated with 50 mM Tris–HCl buffer (pH 7.6). Then, 4 mL of the MCF7 culture supernatant was injected into the column at a flow rate of 1 mL/min. After washing with 50 mM Tris–HCl buffer (pH 7.6), 1-mL fractions containing sEVs were obtained by a linear gradient of 6 mL of 1 M NaCl in 50 mM Tris–HCl buffer (pH 7.6).

### Measurement of sEVs using CD9/CD63 sandwich ELISA

The International Society for Extracellular Vesicles has released a position statement allowing the use of ELISA with antibodies against tetraspanins (CD9, CD63, and/or CD81) as a method for quantifying sEVs (Théry et al. 2018). In this study, sEVs were quantified by sandwich ELISA using anti-CD9 and -CD63 antibodies (Ishida et al. 2020). First, the anti-CD9 antibody (Cosmo Bio Co., Tokyo, Japan) was diluted 500-fold with 10 mM sodium carbonate buffer (pH 9.4). Then, 60 µL of the diluted antibody was added to a plate (MS-8508 M, Sumitomo Bakelite, Tokyo, Japan), which was sealed and allowed to stand overnight at 4 °C. After fixing the anti-CD9 antibody, 300 µL of a TBS-T buffer (25 mM Tris–HCl [pH 7.4], 137 mM NaCl, 2.68 mM KCl, 0.005% Tween 20) was added to each well and washed twice. After washing, 200 µL of TBS-T buffer containing 2% fatty acid-free BSA (Fujifilm, Japan) was added to each well and kept at 37 °C for 1 h. The solution was discarded, and the plate was used as one coated with CD9 antibody. Then, 100 µL of samples diluted with TBS-T containing 2% BSA was added to the plates and incubated for 2 h. The plates were washed three times with 300 µL of TBS-T buffer. After washing, 100 µL of biotinylated anti-CD63 antibody (1:10,000 [019–27713, Wako]) was added to each well and incubated for 1 h. The plates were washed once with 300 µL of TBS-T buffer, and then 100 µL of horseradish peroxidase-conjugated streptavidin (1:10,000 [21130, Thermo Fisher Scientific]) was added and incubated for 30 min. After washing three times with 300 µL of TBS-T buffer, 100 µL of chromogenic substrate solution (Promega, Madison, WI, USA) was added and incubated for 10 min. The reaction was stopped with 50 µL of 1 N HCl, and absorbance at 450 nm was measured. A calibration curve was developed using different amounts of a CD9-CD63 fusion protein included in the CD9/CD63 ELISA kit (Cosmo Bio). Subsequently, sEVs prepared by the UC method were quantified using this calibration curve and used as a standard in subsequent experiments. The concentrations of sEVs in the samples were calculated using this standard and converted as an equivalent to the CD9-CD63 fusion protein. The total protein concentration was measured using the Micro-BCA protein assay (Thermo Fisher Scientific). The purity of sEVs was expressed as CD9/CD63 fusion protein per total protein.

### Western blotting

Fractions containing sEVs were mixed with 4 × SDS sample buffer (0.2 M Tris–HCl [pH 6.8] containing 8% SDS, 40% glycerol, and 0.4% bromophenol blue) and incubated at 37 °C for 30 min prior to electrophoresis (for CD9 and CD63 detection). For TSG101 and Calnexin detection, samples were mixed with 4 × SDS sample buffer containing 2-mercaptoethanol and incubated at 65 °C for 5 min prior to electrophoresis. MCF7 cells were disrupted by ultrasonication (Branson Ultrasonics, Brookfield, CT, USA) and used as a cell lysate. These samples were subjected to SDS–polyacrylamide gel electrophoresis and immunoblotting was performed using the following antibodies: anti-human CD9 (1:2000, clone 12A12, Cosmo Bio), anti-human CD63 (1:2000, clone 3–13, Wako), anti-human TSG101 (1:1000, ab30871, Abcam, Cambridge, UK), or anti-human Calnexin (1:2000, ab22595, Abcam). HRP-conjugated anti-mouse IgG antibody (1:10,000, Abcam) and HRP-conjugated anti-rabbit IgG antibody (1:10,000, Abcam) were used as secondary antibodies. Immunoreactive proteins were detected using an EzWestBlue substrate (ATTO, Tokyo, Japan).

### Transmission electron microscopy (TEM)

Fractions containing sEVs were added onto 150-mesh carbon-film grids (STEM, Tokyo, Japan). The grids were dried with filter paper and stained with 2% uranyl acetate in double-distilled water for 30 s. After the stain was completed, the grids were washed with distilled water and air-dried. sEVs were visualized under JEM-1400 TEM (JEOL, Tokyo, Japan) operating at 80 kV.

## Results

### Affinity of ExoPUA column for phospholipid membranes

**Fig. 1:**
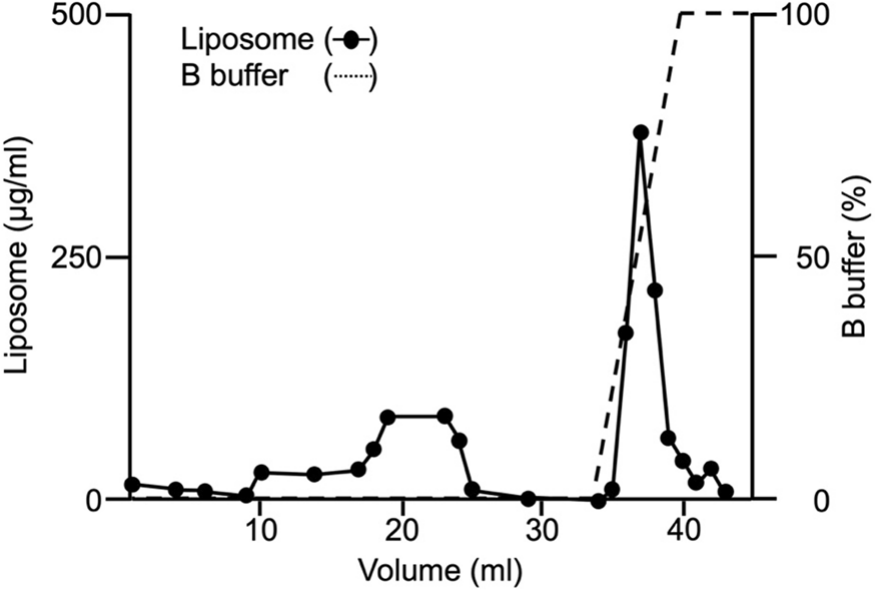
Binding and elution of liposomes using the ExoPUA column. After liposomes were overloaded on the ExoPUA column, liposomes (closed circles) were eluted by a linear gradient of 1 M NaCl (dashed line). One-milliliter fractions containing liposomes were obtained. The concentration of liposomes was measured by absorbance at 220 nm

Prior to sEV purification, we tested whether the ExoPUA column resin, modified with peptides consisting of 25–35 lysine residues, has an affinity for phospholipid membranes. Liposomes comprising DPPC, DPPS, and cholesterol (5:1:4 molar ratio), with a diameter of approximately 130 nm and at a concentration of 79 µg/mL, were applied to the 1-mL ExoPUA column connected to a fast-performance liquid chromatography system (ÄKTA Pure Protein Purification System) (Fig. 1). Liposomes in the flow-through fraction of the first 10 mL were < 15 µg/mL, suggesting that most liposomes were bound to the column. When the next 13 mL flowed through, liposomes partially passed through the column, suggesting that liposomes were overloaded on the column. By the linear gradient of 1 M NaCl, liposomes (total 0.9 ± 0.05 mg in triplicated experiments) were eluted from the 1-mL ExoPUA column, revealing that the maximum binding was approximately 0.9 mg of phospholipid liposomes/1 mL ExoPUA resin.

### Purification of sEVs from the culture supernatant using the ExoPUA column

**Fig. 2:**
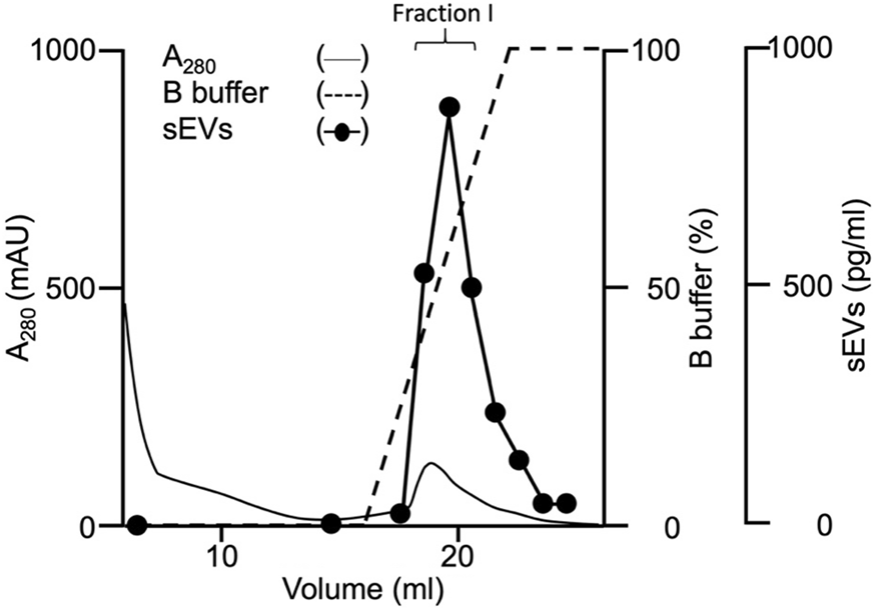
Purification of small extracellular vesicles (sEVs) from the MCF7 culture supernatant. The MCF7 culture supernatant was applied to the ExoPUA column. The column was washed until A_280_ (thin solid line) reached baseline. The sEVs (closed circles) were eluted using the linear gradient of 1 M NaCl (dashed line). One-milliliter fractions containing sEVs were obtained

To demonstrate sEV purification, 4 mL of the MCF7 culture supernatant that was filtered through a 0.2-µm membrane was applied to the 1-mL ExoPUA column connected to the ÄKTA system. All experiments in this study were performed using a 1-mL ExoPUA column. Most proteins flowed through the column during the washing step, and the sEVs were then eluted with a linear gradient of 1 M NaCl (Fig. 2), quantified using the CD9/CD63 sandwich ELISA, as CD9 and CD63 are known to be commonly expressed on the outer membranes of sEVs (Théry et al. 2018; Verweij et al. 2021), and calculated as an equivalent to the CD9-CD63 fusion protein (Ishida et al. 2020). Moreover, sEVs were eluted from the column with a buffer containing approximately 350 mM NaCl.

**Fig. 3:**
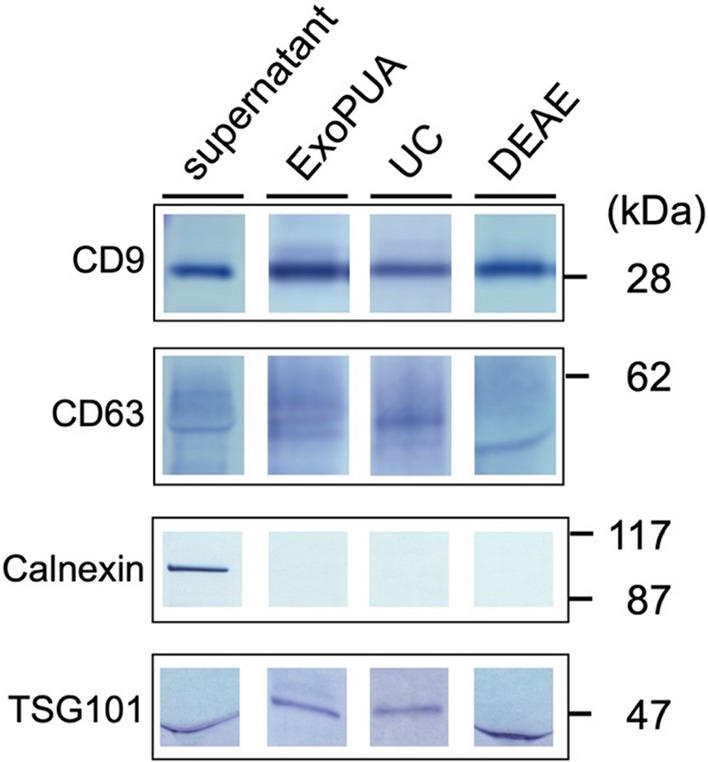
Western blotting of purified small extracellular vesicles (sEVs). Western blotting was performed on the MCF7 culture supernatant (supernatant), sEV fractions purified with the ExoPUA column (ExoPUA), the ultracentrifugation method (UC), and DEAE ionic exchange chromatography (DEAE) using anti-CD9, CD63, Calnexin, and TSG101 antibodies

**Fig. 4:**
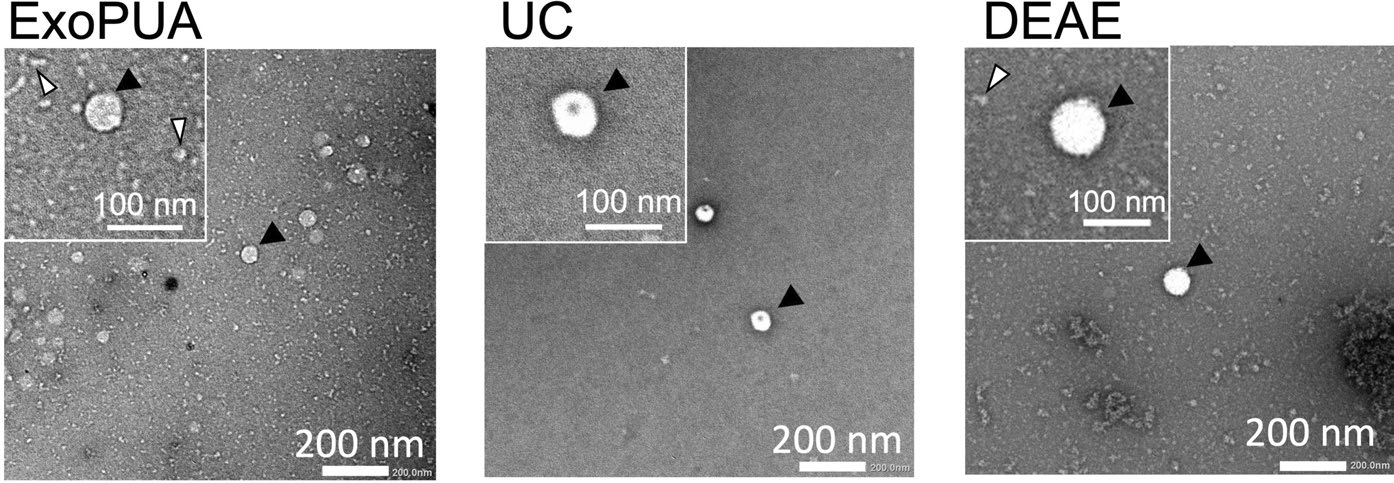
Electron microscopy of small extracellular vesicles (sEVs) purified with ExoPUA column (ExoPUA), the ultracentrifugation method (UC), and DEAE ionic exchange chromatography (DEAE). Purified sEVs were visualized under TEM (magnification: × 20,000; scale bar = 200 nm). Inserts showed sEVs (closed arrowheads) that were digitally enlarged (scale bar = 100 nm). Open arrowheads indicated nanoparticles (< 30 nm)

Fractions from the top three highest sEV concentrations were combined, and sEV purity and yield in the resulting fraction (Fraction I) were calculated. The purity of sEVs was 28 ± 8 pg of CD9-CD63/µg of the total proteins and increased by 58 ± 11-fold compared to that of the MCF7 culture supernatant in triplicate experiments (Table 1). The yield of sEV purification was 52 ± 7%. The purified sEVs contained CD9, CD63, and another marker protein (TSG101), but not the endoplasmic reticulum protein Calnexin (negative control) (Fig. 3). We measured miR-21 which is known to be enriched in cancer sEVs fraction (Melo et al. 2014). Quantitative PCR analysis showed approximately two fewer Ct values by the ExoPUA method than by the UC method, indicating that about four times more miR-21 were isolated (Supplementary Fig. S1). Purified sEVs were observed under TEM; most of the sEVs had a typical round shape (Fig. 4) as did those obtained using the UC method, suggesting that sEVs purified using the ExoPUA column were intact.

One advantage of bind-elute chromatography is scalability. The sample volume of the culture supernatant was increased from 4 to 25 mL and applied to the 1-mL ExoPUA column. Then, sEVs were eluted as described above. The purity and yield of sEVs in fractions (Fraction I) from the top three highest sEV concentrations were 47 ± 15-fold and 32 ± 9% in triplicate experiments, respectively. Although the performance was reduced (19% and 38% reduction in purity and yield, respectively), the culture supernatants of at least 25 volumes of the ExoPUA column were applicable, indicating that sEVs could be purified in the bind-elute mode.

### Comparison of sEV purification with other methods

**Fig. 5:**
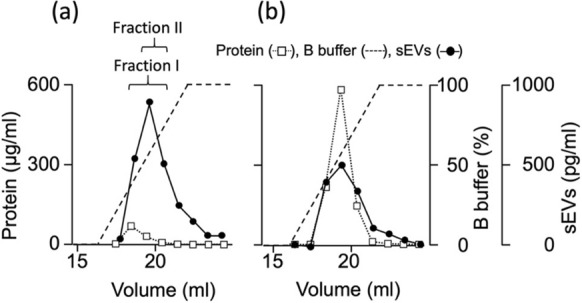
Comparison of sEV purification using ExoPUA and DEAE column chromatography. The MCF7 culture supernatant was applied to the ExoPUA (**a**) and DEAE (**b**) columns. The column was washed until A_280_ reached baseline. Small extracellular vehicles (sEVs; closed circles) were eluted using the linear gradient of 1 M NaCl (dashed line). One-milliliter fractions containing sEVs were obtained. Protein concentrations (open squares) were measured using BCA protein assay

The purity and yield of sEVs isolated from the MCF7 culture supernatant using the UC method were 113 ± twofold and 9 ± 2% in triplicate experiments (Table 1), respectively, indicating that the purity in Fraction I was lower than that in the standard method, although the yield of sEVs was higher. In Fraction I, fractions from the top three highest sEV concentrations were collected. However, the peak fractions of proteins and sEVs were different (Fig. 5a), indicating that the first fraction of Fraction I may contain relatively higher amounts of impurity proteins. Therefore, we omitted the first fraction from the top three highest sEV concentrations, named Fraction II, and recalculated the performance. The purity and yield of sEVs of Fraction II were 82 ± 16-fold and 38 ± 5%, respectively (Table 1). Thus, the purity obtained approaches that of the standard UC method, despite the yield remaining higher.

**Table 1:** Comparison of purification performance

	Supernatant	ExoPUA (Fraction I)	ExoPUA (Fraction II)	UC	DEAE
Yield (%)	100	52 ± 7	38 ± 5	9 ± 2	37 ± 2
Purity (fold)	1.0	58 ± 11	82 ± 16	113 ± 2	3 ± 0.03

*UC* ultracentrifugation, *DEAE* Diethylaminoethyl

Ion exchange chromatography is widely used for the separation of differentially charged molecules. Kosanović et al. (2017) demonstrated sEV purification using DEAE ion exchange chromatography. The supernatant was applied to a 1-mL DEAE column, and sEVs were eluted similarly by a linear gradient of 1 M NaCl (Fig. 5b). The purity and yield of sEVs isolated using DEAE were 3 ± 0.03-fold and 37 ± 2% in triplicated experiments, respectively (Table 1). As many proteins other than sEVs contaminated the sEV fractions, the purity was significantly lower than that with the ExoPUA system.

In addition, nanoparticles smaller than 30 nm were relatively enriched in the purified fractions with ExoPUA and DEAE compared to those purified with the UC method (Fig. 4, open arrowheads). These nanoparticles might require a longer-term or higher-speed centrifugation for their sedimentation. Zhang et al. observed a relatively abundant population of exomeres, measuring approximately 35 nm, in a culture supernatant (Zhang et al. 2018). Recently, Zhang et al. identified an additional population of nanoparticles in the supernatant fraction after the isolation of exomeres by high-speed ultracentrifugation. The additional nanoparticles, termed supermeres, are morphologically distinct from exomeres and display a markedly greater uptake in vivo compared to sEVs and exomeres (Zhang et al. 2021). However, to date, we have not yet determined whether these nanoparticles isolated with ExoPUA are identical to exomeres and/or supermeres.

### Refreshment and sterilization of the ExoPUA column

NaOH is widely accepted for cleaning and sanitization of chromatography resins and systems. It removes proteins and nucleic acids from the column and inactivates most viruses, bacteria, yeasts, fungi, and endotoxins (Jagschies et al. 2007). To refresh and sterilize the ExoPUA column, 0.2 M NaOH was applied and kept overnight at room temperature and then washed with 20% ethanol. The yield of purified sEVs was slightly reduced but remained above > 40% after cleaning and sanitizing the ExoPUA column four times (Supplementary Fig. S2), suggesting that the ExoPUA column can be used repeatedly without significant decreases in sEV yield.

## Discussion

The use of sEVs continues to gain interest owing to their therapeutic potential as drug delivery platforms (Bruno et al. 2009; Colao et al. 2018; Doeppner et al. 2015; Herrmann et al. 2021; Kadota et al. 2021; Kakizaki et al. 2021; Lai et al. 2010; Takeuchi et al. 2021; Zhang et al. 2020). However, a standardized method for sEV purification has yet to be established in the laboratory and industry (Colao et al. 2018; Xu et al. 2016). In the present study, using the ExoPUA protocol (Fraction II) with a linear NaCl gradient elution, we demonstrated an average sEV yield from the culture supernatant of 38%, which is higher than that of the standard UC method (9%). The yield of EVs from culture media by the standard UC method has been reported as 5–25% (Lamparski et al. 2002). The discrepancy in the efficiency of EV isolation has been explained by another group; Nakai et al. (2016) described that the amount of collected sEVs is not consistent due to small and fragile pellets, which can easily be lost during the decantation step, or poor sedimentation efficiency. On the other hand, the use of ExoPUA resulted in an 82-fold increase in purity from the culture supernatant, comparable to the UC method. This study also provided evidence that sEVs remained undamaged following the ExoPUA purification. TEM showed that the sEV size was appropriate, and there were no major differences in shape between sEVs in the supernatant and purified fraction. Additionally, the TSG101 signal observed by western blotting further suggested that the sEV components were retained, as this marker was present within the cytosol of the sEVs (Yoshioka et al. 2013). Therefore, we believe that the ExoPUA-purified sEVs could be similarly used as UC-purified sEVs for both diagnostic testing and pre-clinical trials.

The maximum binding of sEVs remains undetermined but can be deduced from the phospholipid liposome experiment, in which the maximum binding of 130-nm liposomes was approximately 0.9 mg/mL ExoPUA resin. As the dimensions of the phospholipid liposomes are similar to those of sEVs, we expect that the amount of sEVs applicable to the column would be similar. As a methodology, at least 25 volumes of the sample were applicable to the ExoPUA column, indicating that sEVs could be purified in the bind-elute mode. The ExoPUA protocol is a rapid and efficient one-step sEV purification method that may be scaled up in the bind-elute mode.

In the present study, we used the ExoPUA column resin modified with peptides consisting of 25–35 lysine residues. Previously, we reported that peptides comprising 8–16 lysine residues can bind sufficiently to phosphatidylethanolamine-, phosphatidylserine-, and phosphatidylinositol-immobilized plates, but relatively weakly to phosphatidylcholine-immobilized ones (Ishida et al. 2020). The affinity of lysine peptides for lipid membranes can be attributed to their ability to form hydrogen bonds with several phosphate groups in neighboring phospholipid molecules (Ishida et al. 2020). Although sEVs could also be purified using the anion exchange DEAE chromatography method, the purity was significantly lower than that of the ExoPUA protocol owing to the contamination of many other proteins in the elution fractions (Fig. 5b). Therefore, it is likely that the lysine peptides of ExoPUA act as an affinity ligand for phospholipid membranes, rather than as a simple anion exchanger.

The purity of sEVs is essential for accurate research outcomes and for developing safe sEV-based products. Currently, we cannot exclude the possibility that sEV fractions purified using the ExoPUA protocol contain impurity proteins. Thus, more detailed proteomic analysis and molecular profiling of elution peaks, with a focus on lysine peptide-binding proteins, are needed to establish whether this protocol is truly isolating only sEVs. Barnes et al. (2022) have developed a scalable purification method for sEVs using heparin-affinity chromatography (HAC). Heparin is a highly negatively charged glycosaminoglycan known to bind to a wide range of ligands, many of which could be present on sEVs. As affinity ligands of ExoPUA and HAC have opposite charges, the consecutive use of the two columns may allow further purification, resulting in increased sEV purity.

Cleaning, repeated use, and sanitization of chromatography resins are essential for the industry. Protein ligands on affinity resins for the capture of sEVs have been reported using antibodies (Wang et al. 2021) and Tim4 (Nakai et al. 2016), which bind to phosphatidylserine in sEV membranes in a Ca^2+^-dependent manner and can be dissociated in the presence of EDTA. Protein ligands are sensitive to harsh cleaning conditions, such as high NaOH concentrations (Grönberg et al. 2011). In contrast, we demonstrated that the lysine peptides of the ExoPUA column showed resistance to such harsh conditions. Facilities used to manufacture sEVs using the ExoPUA column can be refreshed and sterilized with NaOH, which would comply with industrial standards and good manufacturing practices. In conclusion, the ExoPUA protocol enables multiple sEV purification in a scalable and industrial manner.

### Supplementary Information

Below is the link to the electronic supplementary material.

Supplementary file1 (DOCX 220 KB)

## Data Availability

Correspondence and requests for materials should be addressed to Akio Kuroda.
