# Supplementary material for: Chromatographic purification of small extracellular vesicles using an affinity column for phospholipid membranes
Source: Biotechnol Lett. 2023 Oct 3;45(11-12):1457–66. doi: 10.1007/s10529-023-03430-7 (PMC10635940; doi:10.1007/s10529-023-03430-7)
Supplement: Supplementary file 1 — Supplementary file1 (DOCX 220 KB) [file 10529_2023_3430_MOESM1_ESM.docx]

**Supplementary Information**


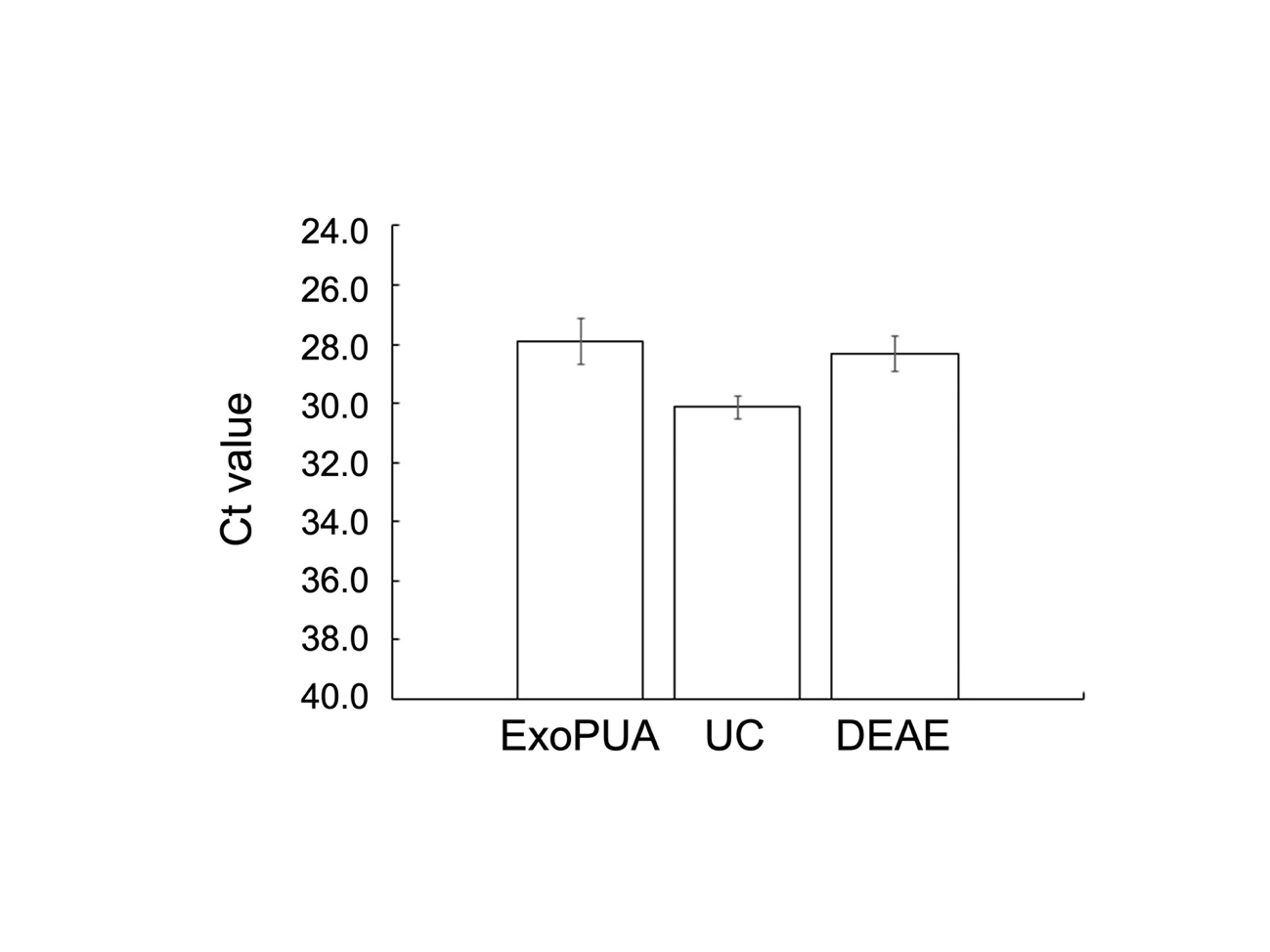
**Supplementary Fig. S1 Quantitative PCR analysis of miR-21 in small extracellular vesicle (sEV) fractions purified by each method.** RNAs were extracted from sEVs fractions purified by each method and reverse transcribed to cDNA using TaqMan MicroRNA Reverse Transcription kit and stem-loop primers (Thermo Fisher Scientific). Quantitative PCR of miR-21 was performed in triplicate with TaqMan MicroRNA assay (has-miR-21, 000397) and TaqMan Fast Advanced Master Mix on a QuantStudio™ 3 real-time PCR system (Thermo Fisher Scientific). Negative control showed a Ct value of >40.


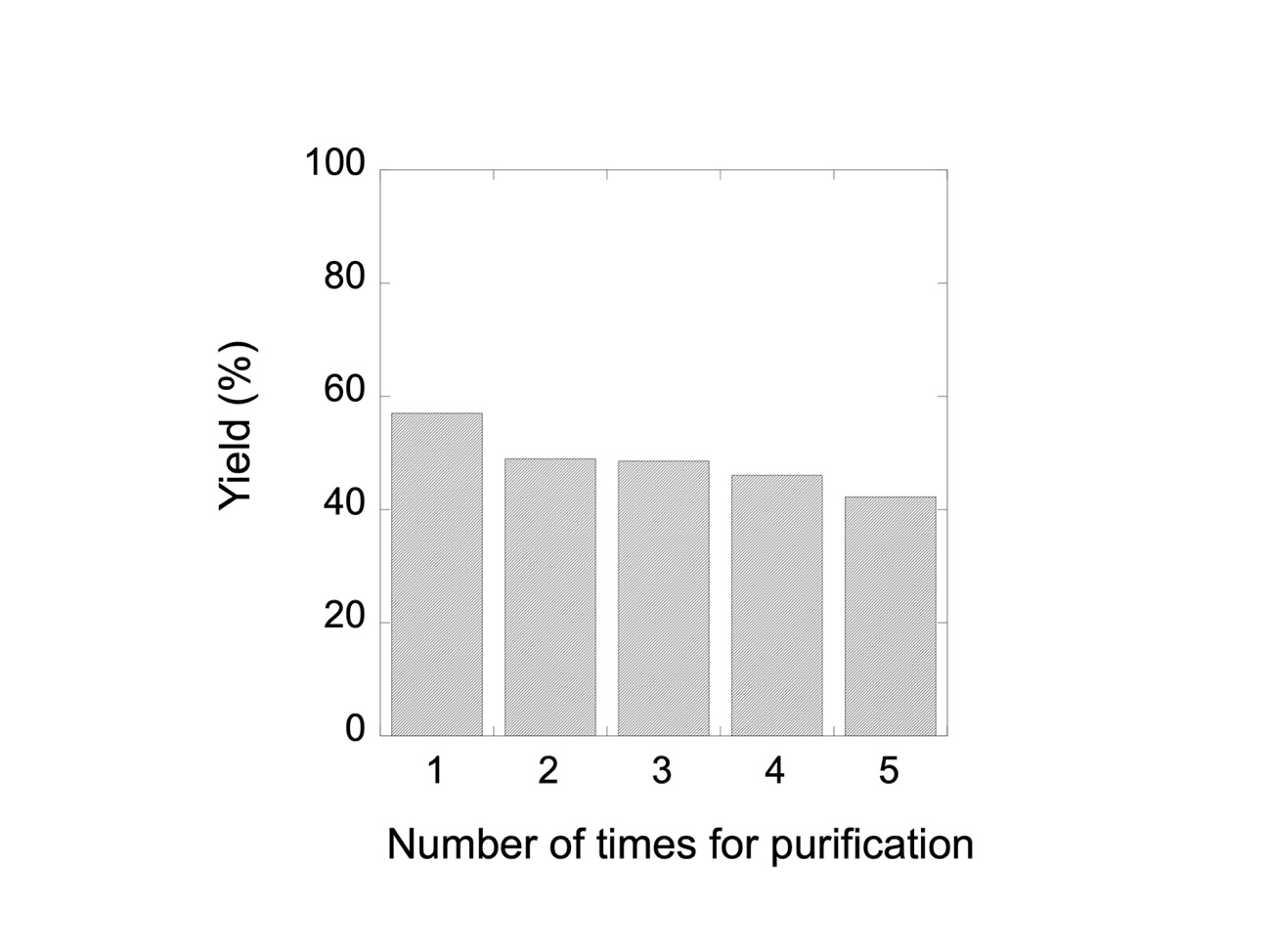


**Supplementary Fig. S2 Performance of the ExoPUA column after cleaning and sanitization of the column.** The yield of small extracellular vesicles (sEVs) isolated from the MCF7 culture supernatant was measured using the ExoPUA protocol (Fraction I). To refresh and sterilize the ExoPUA column, 0.2 M NaOH was applied and kept overnight at room temperature and then washed with 20% ethanol. Then MCF7 culture supernatant was applied again and the yield of sEVs was measured.
